# Human-Level Differentiation of Medulloblastoma from Pilocytic Astrocytoma: A Real-World Multicenter Pilot Study

**DOI:** 10.3390/cancers16081474

**Published:** 2024-04-11

**Authors:** Benedikt Wiestler, Brigitte Bison, Lars Behrens, Stefanie Tüchert, Marie Metz, Michael Griessmair, Marcus Jakob, Paul-Gerhardt Schlegel, Vera Binder, Irene von Luettichau, Markus Metzler, Pascal Johann, Peter Hau, Michael Frühwald

**Affiliations:** 1 Department of Neuroradiology, School of Medicine and Health, Technical University of Munich, 81675 Munich, Germanymichael.griessmair@tum.de (M.G.); 2 TranslaTUM, Center for Translational Cancer Research, Technical University of Munich, 81675 Munich, Germany; 3 Study Groups on CNS Tumors Within the Bavarian Cancer Research Center (BZKF); 4 KIONET, Kinderonkologisches Netzwerk Bayern; 5 Diagnostic and Interventional Neuroradiology, Faculty of Medicine, University Hospital Augsburg, 86156 Augsburg, Germany; brigitte.bison@uk-augsburg.de (B.B.); lars.behrens@uk-augsburg.de (L.B.); 6 Neuroradiological Reference Center for the Pediatric Brain Tumor (HIT) Studies of the German Society of Pediatric Oncology and Hematology, Faculty of Medicine, University Hospital Augsburg, 86156 Augsburg, Germany; 7 Department of Diagnostic and Interventional Radiology, University Hospital Augsburg, 86156 Augsburg, Germany; 8 Department of Pediatric Hematology, Oncology and Stem Cell Transplantation, University of Regensburg, 93053 Regensburg, Germany; marcus.jakob@ukr.de; 9 Department of Pediatric Hematology, Oncology and Stem Cell Transplantation, University Children’s Hospital Würzburg, 97080 Würzburg, Germany; schlegel_p@ukw.de; 10 Department of Pediatrics, Dr. Von Hauner Children’s Hospital, University Hospital, LMU Munich, 80539 Munich, Germany; vera.binder@med.uni-muenchen.de; 11 Division of Pediatric Hematology and Oncology, Department of Pediatrics, Kinderklinik München Schwabing, Children’s Cancer Research Center, TUM School of Medicine and Health, Technical University of Munich, 80333 Munich, Germany; irene.teichert-vonluettichau@mri.tum.de; 12 Pediatric Oncology and Hematology, Department of Pediatrics and Adolescent Medicine, University Hospital Erlangen, Comprehensive Cancer Center Erlangen-EMN (CCC ER-EMN), 91054 Erlangen, Germany; markus.metzler@uk-erlangen.de; 13 Swabian Children’s Cancer Center, Pediatrics and Adolescent Medicine, University Hospital Augsburg, 86156 Augsburg, Germany; pascal.johann@uk-augsburg.de (P.J.); michael.fruehwald@uk-augsburg.de (M.F.); 14 Department of Neurology and Wilhelm Sander-NeuroOncology Unit, University Hospital Regensburg, 93053 Regensburg, Germany; peter.hau@klinik.uni-regensburg.de

**Keywords:** brain, pediatric brain tumor, MRI, artificial intelligence, deep learning

## Abstract

**Simple Summary:**

Reliable preoperative differentiation of pediatric brain tumors can be challenging. While deep learning models have made significant progress in radiology, their use in pediatric populations is limited, typically through limited data availability. In this proof-of-concept study, we investigated the potential of a deep learning classifier trained on a multicenter data set of 195 children to learn to differentiate between pilocytic astrocytoma and medulloblastoma, the two most common infratentorial pediatric brain tumors, which in general present with overlapping imaging features. Our model is validated against the assessment of five independent readers of varying expertise. The final models performed strongly (AUC 0.986) on the unseen test set, correctly predicting the tumor diagnosis in 62 of 64 patients (97%). Compared to human readers, the classifier performed significantly better than relatively inexperienced readers and was on par with pediatric neuroradiologists with specific expertise in pediatric neuro-oncology. Our work highlights the potential of deep learning even in this challenging population and warrants future studies, including different tumor types and diverse acquisition protocols.

**Abstract:**

Medulloblastoma and pilocytic astrocytoma are the two most common pediatric brain tumors with overlapping imaging features. In this proof-of-concept study, we investigated using a deep learning classifier trained on a multicenter data set to differentiate these tumor types. We developed a patch-based 3D-DenseNet classifier, utilizing automated tumor segmentation. Given the heterogeneity of imaging data (and available sequences), we used all individually available preoperative imaging sequences to make the model robust to varying input. We compared the classifier to diagnostic assessments by five readers with varying experience in pediatric brain tumors. Overall, we included 195 preoperative MRIs from children with medulloblastoma (*n* = 69) or pilocytic astrocytoma (*n* = 126) across six university hospitals. In the 64-patient test set, the DenseNet classifier achieved a high AUC of 0.986, correctly predicting 62/64 (97%) diagnoses. It misclassified one case of each tumor type. Human reader accuracy ranged from 100% (expert neuroradiologist) to 80% (resident). The classifier performed significantly better than relatively inexperienced readers (*p* < 0.05) and was on par with pediatric neuro-oncology experts. Our proof-of-concept study demonstrates a deep learning model based on automated tumor segmentation that can reliably preoperatively differentiate between medulloblastoma and pilocytic astrocytoma, even in heterogeneous data.

## 1. Introduction

Tumors of the CNS constitute the largest group of solid neoplasms in children and adolescents [1]. Medulloblastomas, comprising 15–20% of all CNS tumors, constitute the most common malignant CNS neoplasm in this age group. Low-grade gliomas, however, are by far the most common pediatric CNS tumors, accounting for up to 40% of all CNS tumors in childhood. Among these, pilocytic astrocytomas are the single most common entity in children and young adults [2].

Modern imaging techniques have significantly improved the differentiation of low- and high-grade lesions. Several guidelines providing detailed information for standard imaging approaches are in place [3,4]. Usually, on MRIs, medulloblastomas appear iso- to hypointense on T1w images and the T2w signal is variable and often heterogenous, ranging from hyperintense to hypointense. They show restricted diffusion, (which may help differentiate medulloblastoma from pilocytic astrocytoma) and, depending on the subtype, variable enhancement and edema. Intralesional cysts can be found. MR spectroscopy can depict a high choline peak at 3.2 ppm and a taurine peak at 3.4 ppm [5,6,7]. Nevertheless, especially in very young children, atypical localizations for the respective tumor subtype and in the relapse/recurrence situation, imaging features might be less distinct, and neuropathological diagnosis following neurosurgical interventions remains the mainstay of diagnosis.

Distinguishing medulloblastomas from pilocytic astrocytomas is already preoperatively clinically relevant for planning additional staging diagnostics, such as MRI of the neuroaxis and CSF puncture and therapeutic procedures like the extent of neurosurgical resection. While in both tumors a maximum safe resection is generally advised, for the malignant entity of medulloblastoma it is even more prognostically imperative to completely resect the tumor, as residual tumors have repeatedly been shown to be of prognostic importance [8]. CSF cytology and spinal MRI are required to accurately assess the extent of disease in medulloblastoma (as per Chang stages [9]). Both CSF and spinal MRI analyses are postoperatively at risk for false-positive findings (for example, due to hemorrhage or unspecific postoperative change), and are therefore better scheduled preoperatively than postoperatively [10].

Recently, deep learning has enabled unprecedented advances in how clinicians can use imaging data of CNS tumor patients to improve the diagnosis and prognosis of these patients. Modern algorithms enable accurate volumetric segmentation of gliomas across the clinical course of the disease [11,12], which allows for more objective response assessment [13,14], which is now consequently also codified in the respective diagnostic criteria [15,16,17]. Apart from objective volumetry, segmentation is another basis for subsequent image analysis strategies [18], which have yielded important image-based biomarkers for molecular subtyping [19,20] and the prognostication [21] of gliomas. In pediatric neuro-oncology, fewer studies have used preoperative MRI to predict tumor biology [22,23]. Further, some of these studies were comparatively small (<100 patients), hindering the development (and evaluation) of deep learning classifiers and highlighting the need for multi-centric analysis. 

Here, we aimed to develop a deep learning pipeline for automated tumor segmentation and classification into medulloblastoma and pilocytic astrocytoma in a challenging, multicenter data set with high variability in imaging sequences (and their availability) as a pilot study. We further evaluated this classifier against a group of radiologists with varying expertise in pediatric brain tumor assessment. 

## 2. Materials and Methods

### 2.1. Data Set

The German HIT network for children with tumors of the CNS variation is responsible for the neuroradiology reference evaluations for most patients with CNS tumors recruited to the different clinical trials and registries within the community. As such, most diagnostic images of German patients affected by CNS tumors are remotely and continuously evaluated by the German neuroradiology reference center located in Augsburg. As young adults, including individuals aged up to 21 years, are frequently treated at pediatric sites, the reference network also includes MRI images from this age group. The six University Medical Centers in Bavaria, Germany, are organized within the Bavarian Cancer Research Center (BZKF). The pediatric branch of the BZKF is the KIONET, which comprises the six pediatric hematology oncology units at the six University Centers (Augsburg, Erlangen, TU München, LMU München, Regensburg, and Würzburg). In total, about 400 to 450 of the 2010–2200 yearly diagnosed German children with malignancies are located in Bavaria. Up to 65–70 of these are tumors of the CNS variation.

Most of the imaging studies included were completed according to the reference panel recommendations of the HIT network (with some deviations as detailed below). Patients were treated uniformly according to the different clinical trials initiated by the German HIT network or outside clinical trials.

In general, MR images and, whenever available, CTs of patients with CNS tumors are registered on a common data platform. In general, medical data and images are exchanged and stored in the HIT network via the MDPE (medical data and picture exchange) server. This server is operated by the central data management (ZDM) of the GPOH. The data protection concept of this server allows the use of anonymized data for research purposes. The patients and/or their legal guardians gave written informed consent.

For the purpose of this study, all images of affected patients diagnosed within the previous 10 years within the KIONET were available for analysis in an anonymized way.

### 2.2. Image (Pre)Processing

Available preoperative MR sequences (T1w −/+ contrast, T2w, FLAIR, and ADC maps) were rigidly coregistered and transformed into SRI space [24] using NiftyReg [25]. Following skull-stripping using HD-BET [26], we normalized images into [0; 1] within the brain mask and performed automated tumor segmentation using the ensemble strategy implemented in BraTS.Toolkit [27]. BraTS.Toolkit performs tumor segmentation (into necrosis/cysts, contrast-enhancing tumor, and peritumoral edema) using several top-performing algorithms from the BraTS (Brain Tumor Segmentation) challenge [28] and fuses these candidate segmentations into a single consensus segmentation. For tumor segmentation, four input sequences are necessary (T1w −/+ contrast, T2w, and FLAIR). Missing sequences were imputed using a GAN-based strategy [29]. Note, however, that we used these synthetic images only for segmentation, but not for downstream classification. An attending neuroradiologist with over 10 years of experience in brain tumor imaging (BW) checked all resulting segmentations. From the center of mass of the automatically segmented tumor core (i.e., the union of necrotic/cystic and contrast-enhancing tumor areas), we extracted 96 × 96 × 96 patches for downstream classification.

### 2.3. Model Development

We implemented a DenseNet Deep Learning model [30] to predict the tumor entity. In brief, a DenseNet is characterized by dense connections within a layer, where each block receives direct input from all blocks preceding it. This architecture helps to exploit feature re-use to efficiently learn image features using comparatively small filter banks. The reference implementation of DenseNet121 in Keras (version 2.6; https://www.tensorflow.org/versions/r2.6/api_docs/python/tf/keras/applications/densenet/DenseNet121, accessed on 1 June 2023), which consists of 4 layers with 6, 12, 24, and 16 blocks, respectively, was used for this study. Given the three-dimensional nature of our input images, we changed the architecture to 3D convolutions and pooling operations and switched to a single (binary) output neuron with sigmoid activation.

The input to our network consisted of 64 × 64 × 64 sized patches, where all available imaging sequences were concatenated along the last axis. Missing sequences were replaced using blank masks. During training, we random-cropped the 96 × 96 × 96 patches from above as a data augmentation strategy, while for testing, we center-cropped patches (given that the tumor core’s center of mass is the center of each patch). Besides this random cropping, we also implemented random gamma adjustment, random Gaussian noise, and random axis flipping as intensity or geometric augmentation strategies. In addition, to improve the robustness of our network to missing sequences, we randomly blanked out one input sequence. The network was trained using the Adam optimizer with a base learning rate of 1 × 10^−3^ and cosine annealing schedule, and a batch size of 42 for a total of 250 epochs using binary cross-entropy loss on an Nvidia Quadro RTX 8000 GPU with 48 GB of RAM.

### 2.4. Statistical Evaluation and Comparison

We assessed classifier performance in a hold-out test set (*n* = 64 patients) not used during training. In addition, we provided MR images from these test set patients to five pediatric radiologists and neuroradiologists with varying levels of expertise in pediatric gliomas, asking them to classify tumors as either medulloblastoma or pilocytic astrocytoma. To compare the proportion of samples correctly predicted between the classifier and the human raters, we calculated the *Z* statistic as follows [31]:

$$Z=\frac{p1-p2}{\sqrt{2p\left(1-p\right)/n}}$$

where *p*_1_ is the proportion of the correctly predicted *n* samples for the model (*x*_1_*/n*), *p*_2_ is the respective proportion for the human rater, and *p* is their mean (*(x*_1_ *+ x*_2_)/2 × *n*).

In addition, we plotted the feature representation (from the global average pooling layer) for the test set data after tSNE (T-distributed Stochastic Neighbor Embedding) dimensionality reduction (employing “cosine” distance) using the scikit-learn (version 1.2.2) implementation.

## 3. Results

### 3.1. Patient Characteristics

Our cohort comprised a total of 195 pediatric and adolescent patients with either medulloblastoma (*n* = 69) or pilocytic astrocytoma (*n* = 126). The age distribution was similar in both groups: the median age for patients with medulloblastoma was 8.5 years (interquartile range 4.8–12.9 years) and 9.1 years (interquartile range 4.9–13.5 years) for patients with pilocytic astrocytoma (*p* = 0.93, Mann–Whitney U test). Of all patients, seventeen had a missing ADC map, six patients had missing T2w images, and five patients had no non-enhanced T1w images. Also, with respect to imaging parameters, we observed a high variability across patients. While contrast-enhanced T1w images tended to be acquired in an isotropic fashion with voxel sizes < 2 × 2 × 2 mm^3^, the remaining sequences were mainly acquired in 2D, i.e., with a through-plane resolution usually exceeding 4 mm. We performed a stratified split of this group into a training cohort of 131 patients (*n* = 46 medulloblastomas and *n* = 85 pilocytic astrocytomas) and an independent test cohort of 64 patients (*n* = 23 medulloblastomas and *n* = 41 pilocytic astrocytomas). 

### 3.2. Deep Learning Results

**Figure 1:**
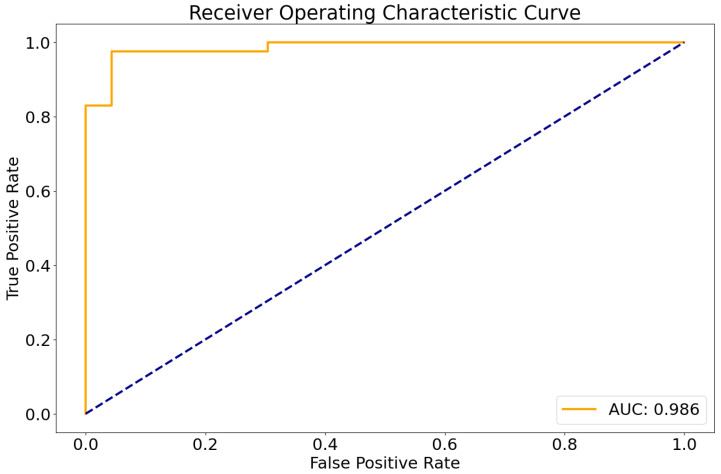
Receiver operating characteristic curve of the DenseNet model in the test set. AUC, Area under the curve.

The entire processing runtime (including registration, skull-stripping, segmentation, and classification) amounted to less than 5 min per sample on a standard workstation with a GPU (12 GB VRAM). In the independent test cohort, the developed classifier showed a very high area under the receiver operating characteristic curve of 0.986 (Figure 1).

**Figure 2:**
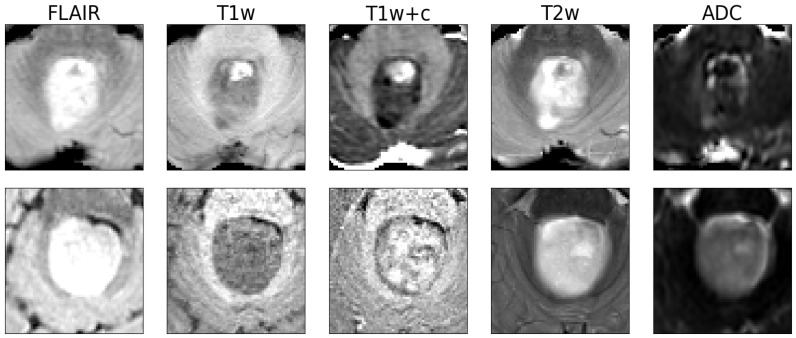
The two examples misclassified by the model. The top row is a medulloblastoma, and the bottom row is a pilocytic astrocytoma. Shown are axial slices of the 64^3^ input patches presented to the network (the human rater received whole-brain 3D volumes).

Using a pre-defined decision threshold of 0.5 to binarize the predictions, 62 out of 64 samples (97%) were correctly classified as either medulloblastoma or pilocytic astrocytoma in comparison to neuropathological diagnosis according to the WHO classification, which was used as the gold standard. The model misclassified one pilocytic astrocytoma and one medulloblastoma. For these two cases, representative central slices are shown in Figure 2. For correctly predicting medulloblastoma, this translated into a sensitivity of 0.96 and a specificity of 0.97. For pilocytic astrocytoma, sensitivity was 0.97 and specificity 0.96, consequently. In total, the resulting classifier had an averaged F1 score of 0.96 and a Matthews correlation coefficient of 0.93.

**Figure 3:**
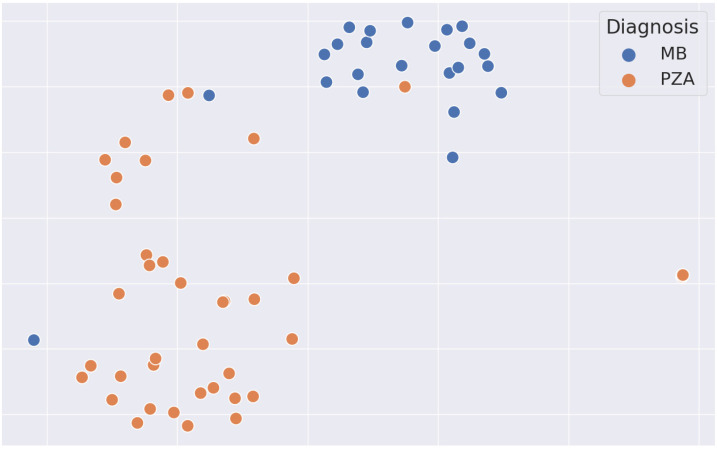
tSNE plot of the learned feature representations in the test set. Note the clear distinction between medulloblastoma (blue) and pilocytic astrocytoma (orange), highlighting the robust separations in the learned representations. tSNE, T-distributed Stochastic Neighbor Embedding; MB, medulloblastoma; PZA, pilocytic astrocytoma.

To investigate the learned representations of the two different tumor types, we additionally plotted the features (taken from the global average pooling layer immediately before the classification head) after dimensionality reduction with tSNE (T-distributed Stochastic Neighbor Embedding), as shown in Figure 3. For the vast majority of samples, the two tumor types show a clearly distinct clustering; only for one case each (which are the two misclassified cases as shown in Figure 2) were the representations not distinctive. 

### 3.3. Sequence Importance

To better understand the importance of the different sequences for tumor classification (and to evaluate the robustness of our model to missing data), we performed an additional experiment where we intentionally blanked out each input sequence (FLAIR, T1w, T1w+c, T2w, and ADC) in turn and re-calculated the AUC for the test set. Coming from an AUC of 0.986 when using all available data, the test set performance remained stably high when omitting ADC (0.983), FLAIR (0.963), T1w (0.984), or even T1w+c (0.987). Only upon the exclusion of T2w images did we observe a noticeable drop in performance (AUC 0.92).

### 3.4. Expert Comparison

To compare our model to human raters, we asked five radiologists with varying levels of expertise in pediatric brain tumor imaging to classify the 64 test set cases. The results are summarized in Table 1. For the two experts from the Neuroradiological Reference Center for the pediatric brain tumor (HIT) studies of the German Society of Pediatric Oncology and Hematology, the classification performance was very similar (in one case identical) to our model. The two expert mistakes were different cases than the classifier. For the remaining three raters, the classification accuracy of our deep learning model was higher, in particular for the two neuroradiological residents with expertise in adult brain tumor imaging but without relevant prior experience in pediatric brain tumors. Here, the proportion of correctly classified cases was significantly higher for the deep learning model compared to both readers (*p* < 0.05 each).

## 4. Discussion

Reliable preoperative differentiation of medulloblastoma and pilocytic astrocytoma can be challenging. Deep learning models may meaningfully support clinicians in this task. However, the development of these tools is typically limited by small sample sizes, particularly in single-center studies [32]. Here, we performed a proof-of-concept study demonstrating how a deep learning pipeline encompassing segmentation and classification can leverage a highly heterogeneous imaging data set to train a reliable classifier with a strong performance (AUC 0.986). We further demonstrate that our model performs rather positively when compared to highly specialized and experienced pediatric neuroradiologists from the Neuroradiological Reference Center for the pediatric brain tumor (HIT) studies of the German Society of Pediatric Oncology and Hematology and outperforms neuroradiology residents. Our results pave the way for larger, multicenter studies, including further pediatric tumor entities, to train generalizable image classifiers for clinical applications.

An increasing number of deep learning-based approaches for tumor detection and segmentation or classification in adult neuro-oncology is on record. At the same time, remarkably fewer studies exist in pediatric neuro-oncology, as highlighted in a recent review by Madhogarhia and colleagues [32]. These authors identify limited sample sizes (particularly in single institutional studies) as one major challenge for developing such models in pediatric patients. Many of the studies in this review contained data sets of fewer than 100 patients, which impairs the training of robust deep learning models. In contrast, we curated a large, heterogeneous data set from the six University Medical Centers in Bavaria, Germany, organized within the Bavarian Cancer Research Center (BZKF). This “exposure” of the DenseNet model during training to a large variety of patients and MR scanners—to account for technical variations—translated into a highly efficient classifier. Notably, the cases the model misclassified are different from the cases in which the human raters erred. This “complementarity” of errors highlights the potential for a high-level joint assessment by a deep learning model and radiologists to correct each other’s mistakes, which would lead to 100% accuracy, at least in our cohort. In addition, deep learning models promise to incorporate additional information, for example, from clinical data, other imaging modalities such as PET or CSF cytology, and genomic analyses, supporting clinicians in diagnostic (and therapeutic) decisions even preoperatively: Tumor resection following neoadjuvant chemotherapy has been a long-standing hope in pediatric neuro-oncology, i.e., the prospect of complete resection of a smaller lesion with the potential of fewer permanent neurological deficits stands out. Knowing in advance the histology (and potentially also the molecular background) of a lesion employing AI imaging in conjunction with liquid biopsy and potentially other imaging technologies such as PET may lead such a project to success.

Some studies into the imaging-based differentiation of pediatric brain tumors investigated the importance of individual MR sequences. Among the commonly acquired sequences, T2w images [33] and ADC maps from diffusion-weighted imaging [34,35] have been identified as particularly helpful in this task. Consequently, we included these sequences wherever available. Upon experimental evaluation of sequence importance (through round-robin omission), we found that T2w stood out for its relevance for correct classification in our setting.

As with any real-world data set, we observed missing (or corrupted, e.g., by motion) sequences. Instead of excluding such cases (as is usually the case), we specifically opted to make our classifier robust to missing data. Handling missing data is an active area of research, and several strategies have been devised to deal with this. With recent developments in generative AI, models have been developed to synthesize missing sequences from existing data. The use of these models has, for example, been demonstrated for tumor segmentation [29], and we subsequently employed this strategy for automated segmentation as well, given the availability of a pre-trained model. For the classification, however, we specifically opted for a different strategy, as to our knowledge, no task-specific generative network is available. Here, we adopted a random drop-out strategy, i.e., we randomly deleted input sequences during training as part of our augmentation pipeline. Recently, such a strategy has also been demonstrated to achieve state-of-the-art results in brain tumor segmentation [36] and provide an attractive additional strong augmentation paradigm for training. Further, when including multiple input sequences, care must be taken not to overfit the classifier by adding excessive imaging “noise”, with potentially harmful consequences for generalizability [37]. The random drop-out strategy we employed provides additional regularization to avoid overfitting.

In a prior study, Zhou et al. developed a machine learning classifier to differentiate pediatric posterior fossa tumors on MRIs [38]. They report high AUC values for the classification of medulloblastoma and pilocytic astrocytoma. Similar to our findings, they report that non-expert radiologists have lower accuracy than their classifier model. A key difference to our model is their reliance on hand-crafted radiomics features extracted from manually drawn tumor masks: prior studies have shown that differences in segmentation critically affect the feature stability and hence, the reproducibility of results [39]. We thus chose a patch-based approach (centered around the center of mass of the tumor segmentation) paired with an automatic segmentation module, enabling a fully-automated image analysis without the need for manual interference. Coupled with our random cropping augmentation, our model should, therefore, be robust against minor differences in seed voxel location, i.e., as long as the seed voxel is placed well inside the tumor, downstream classification stability should not be affected. This robustness against input variations is a strength of our approach.

Quon et al. report on developing a 2D slice-wise deep learning classifier for differentiating medulloblastoma, pilocytic astrocytoma, diffuse midline glioma, and ependymoma in a large cohort of 617 children [40]. Their final ensemble classifier had an F1 score of 0.8, albeit in a more challenging multi-class classification. Again, in line with our results, they found that when comparing model performance with radiologists, in particular, less experienced readers have lower diagnostic accuracy. As opposed to our work, only axial T2w slices were used due to the 2D slice-wise design. Conceptually, our 3D concatenation approach allows the deep learning model to capture relevant synergies between the different modalities and offers another explanation for the higher performance we observe in our model. This improved differentiation of medulloblastoma and pilocytic astrocytoma can also be seen when comparing the representations in feature space. tSNE plotting shows a clear separation of the two tumor types, where medulloblastomas cluster particularly tightly.

We acknowledge some limitations of our work. First, our analysis focused on medulloblastoma and pilocytic astrocytoma, excluding other entities such as ependymoma or diffuse midline glioma. While these are the two most common infratentorial tumors in children, and their preoperative differentiation has clinical relevance, this creates a specific context for this study and clearly labels our study an experimental proof-of-concept study. Also, owing to the retrospective nature of this study, with several cases diagnosed in the early 2010s, molecular diagnoses as outlined in the 2021 WHO classification of brain tumors (CNS5) [41] were only available for some cases. This precluded training classifiers for molecular alterations (such as BRAF for pilocytic astrocytomas or the medulloblastoma subgroups [22]), which, of course, is an attractive future research direction given the importance of these markers for diagnosis and also, in part, therapeutic stratification. Second, segmentation and downstream patch-based classification are separate tasks in our pipeline. Despite efforts to improve segmentation, particularly for pediatric brain tumors in the 2023 BraTS challenge [42], jointly optimizing both tasks holds promise for further improving performance. With the broader availability of manually annotated pediatric brain tumor data sets, end-to-end optimized deep learning approaches for joint segmentation/detection and classification of pediatric brain tumors are therefore an attractive follow-up extension to our study. Lastly, when considering the possibilities of joint human and AI assessment of pediatric brain tumors, explainable AI strategies are another attractive avenue of research. Recent advances in joint learning representations from text (e.g., radiology reports) and medical images, such as BioMedCLIP [43], offer intriguing opportunities for improving the interaction between radiologists and deep learning models: by allowing the latter to offer textual explanation, the otherwise seemingly intractable decision made by a deep learning classifier can be retraced by a radiologist.

## 5. Conclusions

In summary, we developed and validated a robust deep learning model for the automated precise differentiation of medulloblastoma from pilocytic astrocytoma in a large, heterogeneous data set of pediatric brain tumor patients. Based on our proof-of-concept study, demonstrating how reliable, fully automated classifiers (including tumor segmentation) can be trained from heterogeneous multicenter data, our work highlights the potential of deep learning models to make this expert knowledge broadly available. In future studies, we will implement additional entities and prospective data sets to further validate our approach.

## Figures and Tables

**Table 1:** Results for the DL-based model and the human raters.

	Accuracy	F1	MCC	*
DL Model	0.97	0.96	0.93	
Expert Rater 1	1	1	1	
Expert Rater 2	0.97	0.96	0.93	
Pediatric Radiologist	0.92	0.91	0.82	
Resident 1	0.87	0.86	0.72	*
Resident 2	0.84	0.81	0.66	*

* denotes a significantly (*p* < 0.05) higher accuracy for the DL-based model. MCC, Matthews Correlation Coefficient.

## Data Availability

The data sets presented in this article are not readily available because of ethical and data privacy requirements.
